# Supplementary material for: The NeflE397K mouse model demonstrates muscle pathology and motor function deficits consistent with CMT2E
Source: bioRxiv. 2025 Feb 3:2025.02.02.636119. Preprint. [Version 1] doi: 10.1101/2025.02.02.636119 (PMC11838438; doi:10.1101/2025.02.02.636119)
Supplement: 1 [file NIHPP2025.02.02.636119V1-supplement-1.pdf]

bioRxiv preprint doi: https://doi.org/10.1101/2025.02.02.636119; this version posted February 3, 2025. The copyright holder for this preprint (which was not certified by peer review) is the author/funder, who has granted bioRxiv a license to display the preprint in perpetuity. It is made available under aCC-BY 4.0 International license.

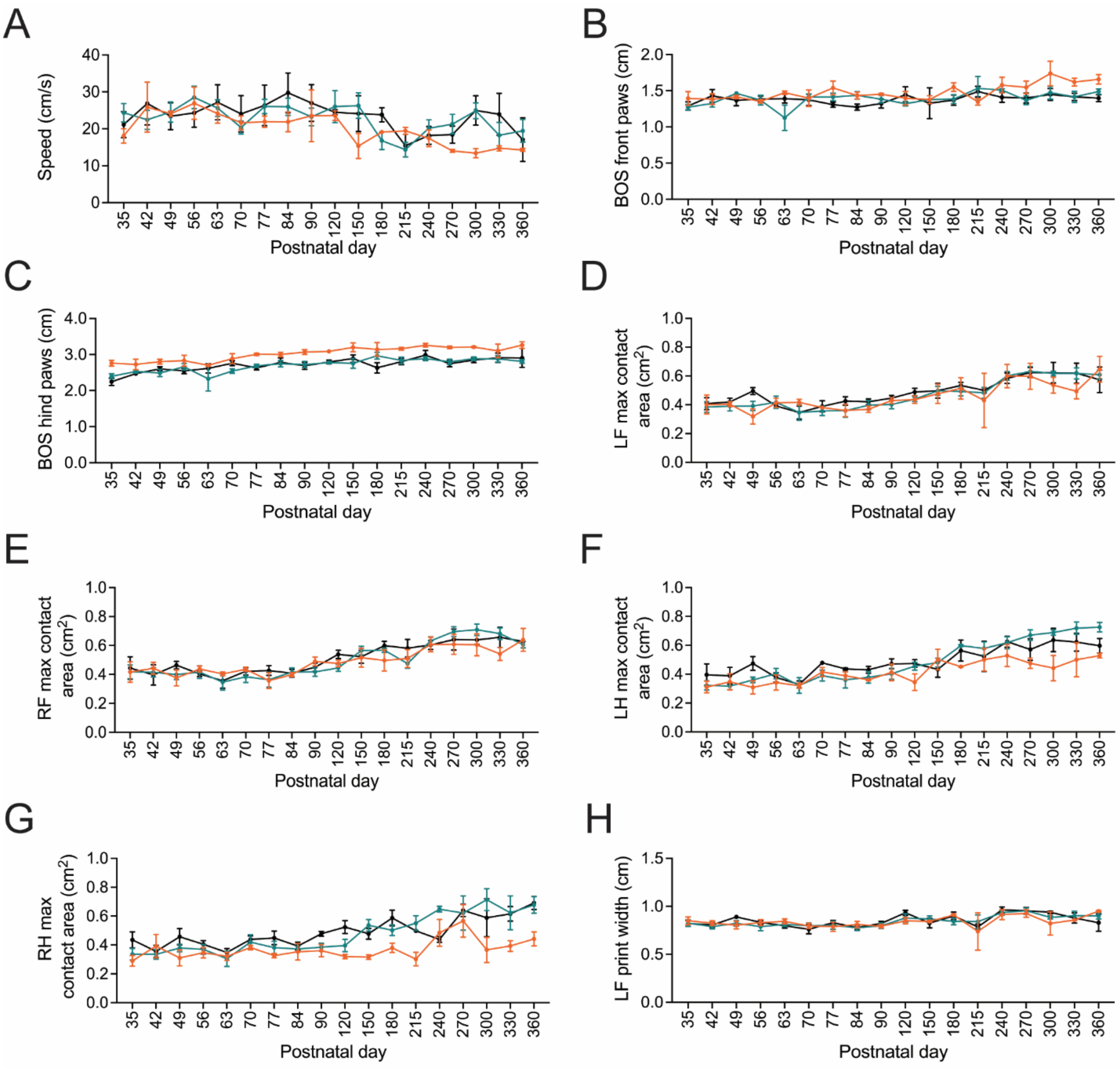

Supplementary Figure S1A-H. CatWalk assessments in *Nefl* mutant mice show differences. Longitudinal CatWalk assessment was performed in *Nefl*$^{+/+}$ (black), *Nefl*$^{+/E397K}$ (teal), and *Nefl*$^{E397K/E397K}$ (orange) mice to evaluate individual footprint-based parameters. (A) Speed measured in centimeters per second (cm/s). (B) Base of support (BOS) of front paws in centimeters (cm). (C) BOS of hind paws in centimeters (cm). (D) Left front (LF) maximal contact area. Maximal contact indicates the moment during a stance phase for which the footprint is largest recorded in centimeters squared. (E) Right front (RF) maximal contact area recorded in centimeters squared. (F) Left hind (LH) maximal contact area recorded in centimeters squared. (G) Right hind (RH) maximal contact area recorded in centimeters squared. (H) Left front (LF) print width in centimeters. Print width Indicates the width of the complete footprint. Mixed-effect analysis with a Bonferroni's multiple comparison test and two-way ANOVA and repeated measures one-way ANOVA with Dunnett multiple comparison test were used to determine significance.

bioRxiv preprint doi: https://doi.org/10.1101/2025.02.02.636119; this version posted February 3, 2025. The copyright holder for this preprint (which was not certified by peer review) is the author/funder, who has granted bioRxiv a license to display the preprint in perpetuity. It is made available under aCC-BY 4.0 International license.

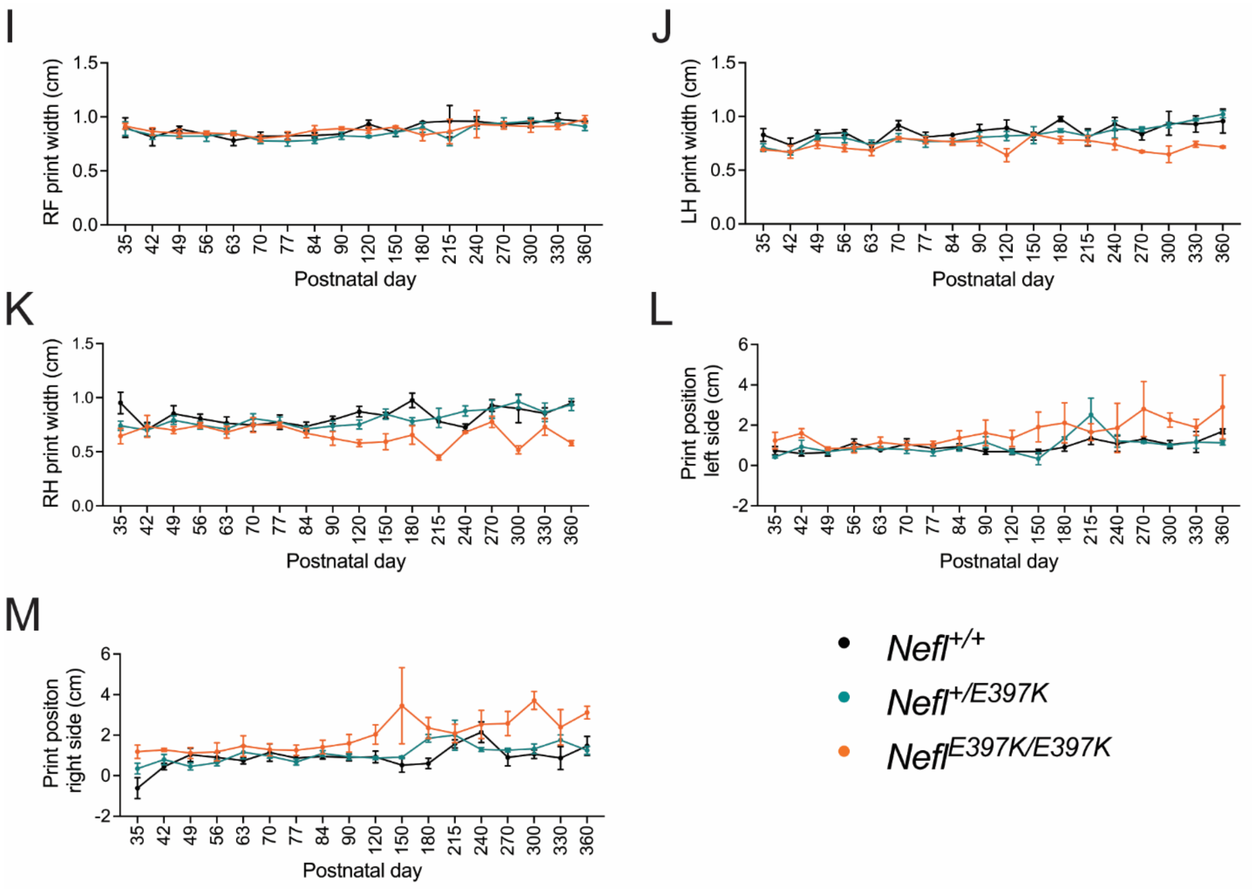

Supplementary Figure S1I-M. CatWalk assessments in *Nefl* mutant mice show differences. Longitudinal CatWalk assessment was performed in *Nefl*$^{+/+}$ (black), *Nefl*$^{+/E397K}$ (teal), and *Nefl*$^{E397K/E397K}$ (orange) mice to evaluate individual footprint-based parameters (I) Right front (RF) print width in centimeters. (J) Left hind (LH) print width in centimeters. (K) Right hind (RH) print width in centimeters. (L) Print position of left side paws. Print position indicates the position of the hind paw relative to the previous position of the fore paw. (M) Print position of right side paws. Mixed-effect analysis with a Bonferroni's multiple comparison test and two-way ANOVA and repeated measures one-way ANOVA with Dunnett multiple comparison test were used to determine significance.

bioRxiv preprint doi: https://doi.org/10.1101/2025.02.02.636119; this version posted February 3, 2025. The copyright holder for this preprint (which was not certified by peer review) is the author/funder, who has granted bioRxiv a license to display the preprint in perpetuity. It is made available under aCC-BY 4.0 International license.

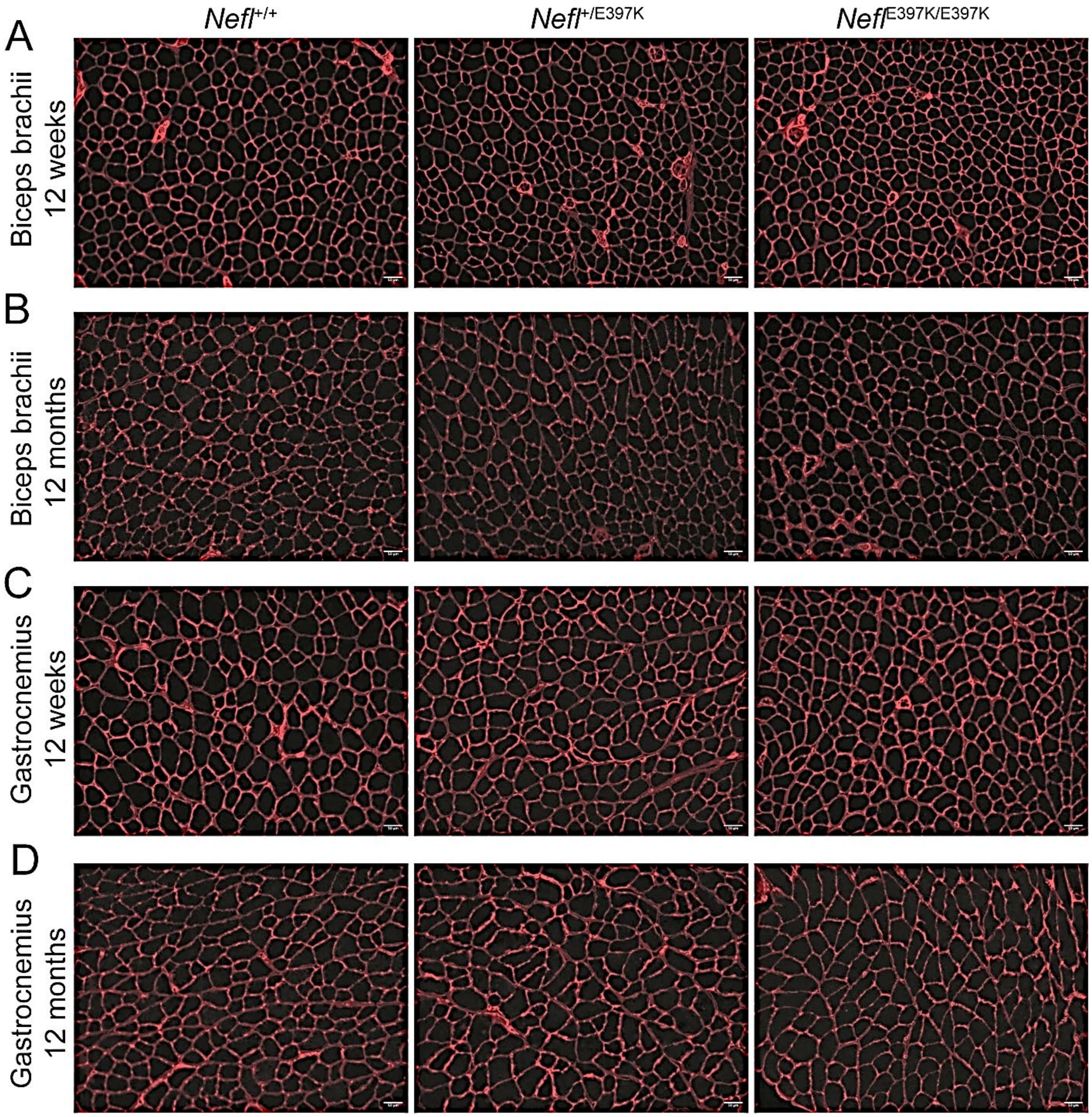

Supplementary Figure S2. Representative muscle fiber images. Representative cross-sectional images of muscle fibers of biceps brachii and gastrocnemius muscles at twelve weeks and twelve months of $Nefl^{+/+}$, $Nefl^{+/E397K}$, and $Nefl^{E397K/E397K}$ mice immunolabeled with laminin. (A) Biceps brachii cross sections of twelve weeks old mice. (B) Biceps brachii cross sections of twelve months mice. (C) Gastrocnemius cross sections of twelve weeks old mice. (D) Gastrocnemius cross sections of twelve months old mice.

bioRxiv preprint doi: https://doi.org/10.1101/2025.02.02.636119; this version posted February 3, 2025. The copyright holder for this preprint (which was not certified by peer review) is the author/funder, who has granted bioRxiv a license to display the preprint in perpetuity. It is made available under aCC-BY 4.0 International license.

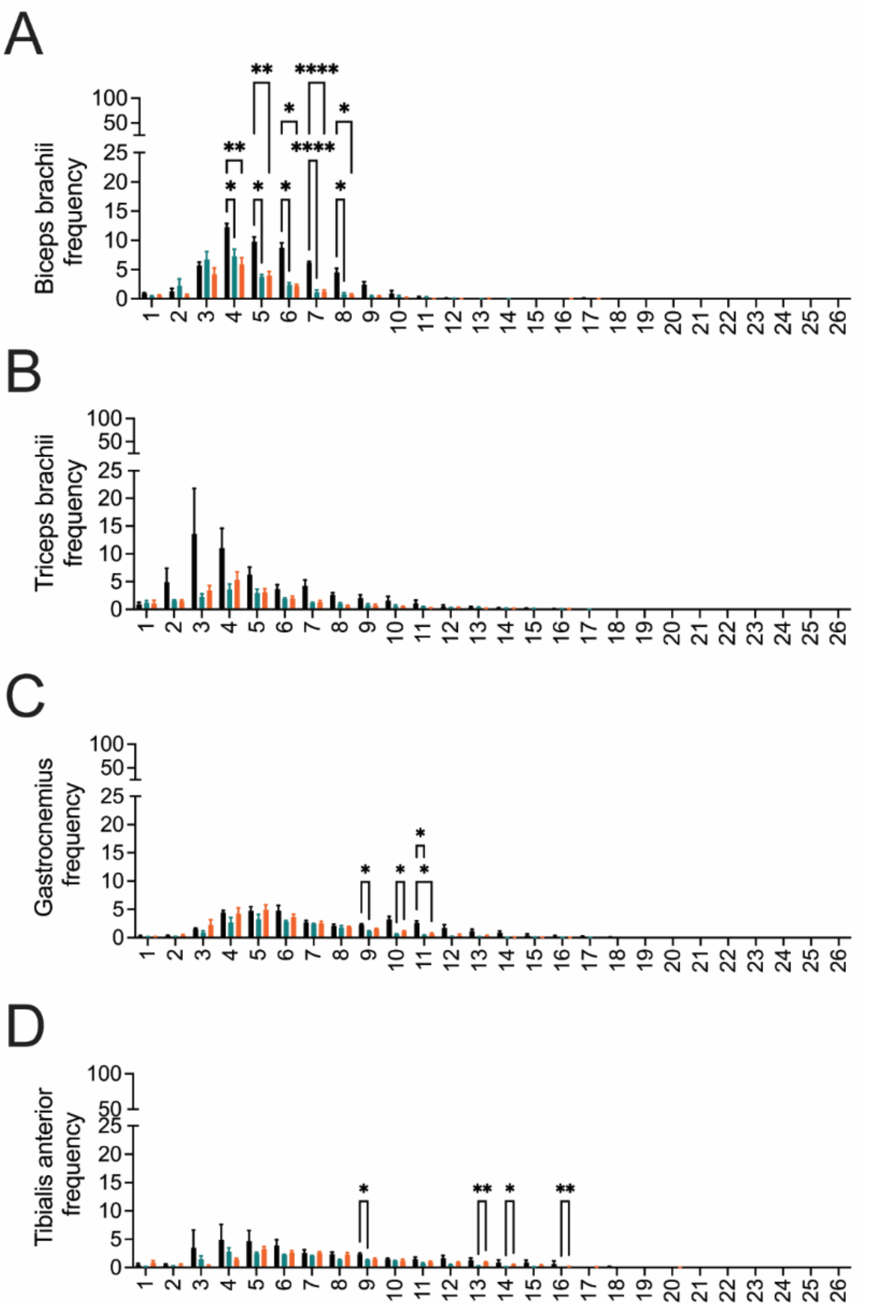

Supplementary Figure S3. *Nefl* mutants show changes in the distribution of muscle fiber size. Muscle fiber area distribution in the forelimbs and hindlimb muscles of *Nefl*$^{+/+}$ (black), *Nefl*$^{+/E397K}$ (teal), and *Nefl*$^{E397K/E397K}$ (orange) mice at twelve weeks. Cross-sections of individual muscle fibers of biceps brachii, triceps brachii, gastrocnemius, and tibialis anterior muscles were binned by area into 400 μm^2 intervals and expressed as percentage of muscle fibers. (A) Biceps brachii muscle fiber area distribution (**P*=0.0434-0.0101, ***P*=0.0076, 0.0072, *****P*<0.0001). (B) Triceps brachii muscle fiber area distribution. (C) Gastrocnemius muscle fiber area distribution (**P*=0.0362-0.222). (D) Tibialis anterior muscle fiber area distribution (**P*=0.0438, 0.0435, ***P*=0.0022, 0.0028). Distribution in μm^2 1=0-400, 2=400-800, 3=800-1200, 4=1200-1600, 5=1600-2000, 6=2000-2400, 7=2400-2800, 8=2800-3200, 9=3200-3600, 10=3600-4000, 11=4000-4400, 12=4400-4800, 13=4800-5200, 14=5200-5600, 15=5600-6000, 16=6000-6400, 17=6400-6800, 18=6800-7200, 19=7200-7600, 20=7600-8000, 21=8000-8400, 22=8400-8800, 23=8800-9200, 24=9200-9600, 25=9600-10000, 26=>10000. Mixed-effect analysis with Tukey's multiple comparison test were used to determine significance.

bioRxiv preprint doi: https://doi.org/10.1101/2025.02.02.636119; this version posted February 3, 2025. The copyright holder for this preprint (which was not certified by peer review) is the author/funder, who has granted bioRxiv a license to display the preprint in perpetuity. It is made available under aCC-BY 4.0 International license.

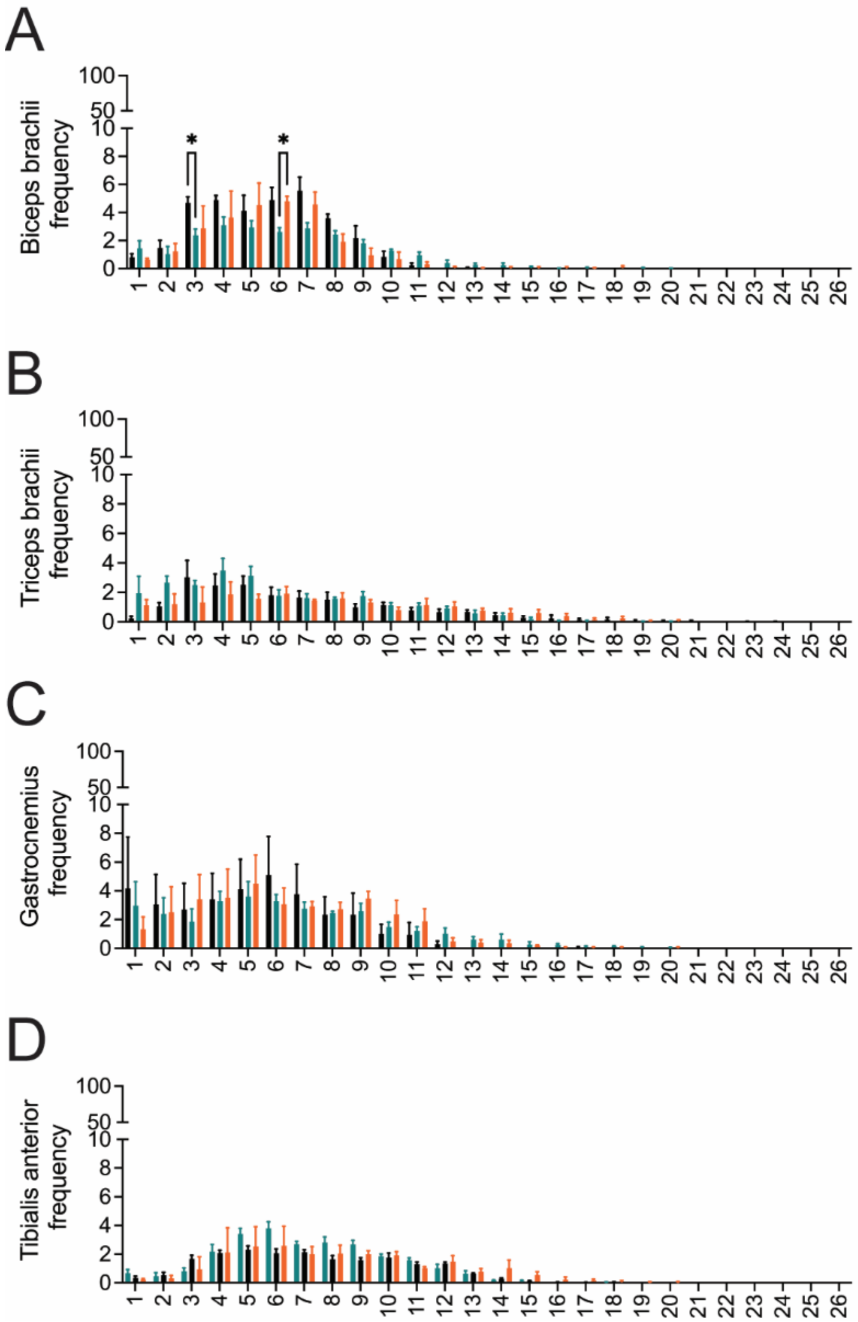

Supplementary Figure S4. The distribution of muscle fiber size at twelve months. Muscle fiber area distribution in the forelimbs and hindlimb muscles of *Nefl*$^{+/+}$ (black), *Nefl*$^{+/E397K}$ (teal), and *Nefl*$^{E397K/E397K}$ (orange) mice at twelve months. Cross-sections of individual muscle fibers of biceps brachii, triceps brachii, gastrocnemius, and tibialis anterior muscles were binned by area into 400 μm^2 intervals and expressed as percentage of muscle fibers. (A) Biceps brachii muscle fiber area distribution (*P=0.0283, 0.0145). (B) Triceps brachii muscle fiber area distribution. (C) Gastrocnemius muscle fiber area distribution. (D) Tibialis anterior muscle fiber area distribution. Distribution in μm^2 1=0-400, 2=400-800, 3=800-1200, 4=1200-1600, 5=1600-2000, 6=2000-2400, 7=2400-2800, 8=2800-3200, 9=3200-3600, 10=3600-4000, 11=4000-4400, 12=4400-4800, 13=4800-5200, 14=5200-5600, 15=5600-6000, 16=6000-6400, 17=6400-6800, 18=6800-7200, 19=7200-7600, 20=7600-8000, 21=8000-8400, 22=8400-8800, 23=8800-9200, 24=9200-9600, 25=9600-10000, 26=>10000. Mixed-effect analysis with Tukey's multiple comparison test were used to determine significance.

bioRxiv preprint doi: https://doi.org/10.1101/2025.02.02.636119; this version posted February 3, 2025. The copyright holder for this preprint (which was not certified by peer review) is the author/funder, who has granted bioRxiv a license to display the preprint in perpetuity. It is made available under aCC-BY 4.0 International license.

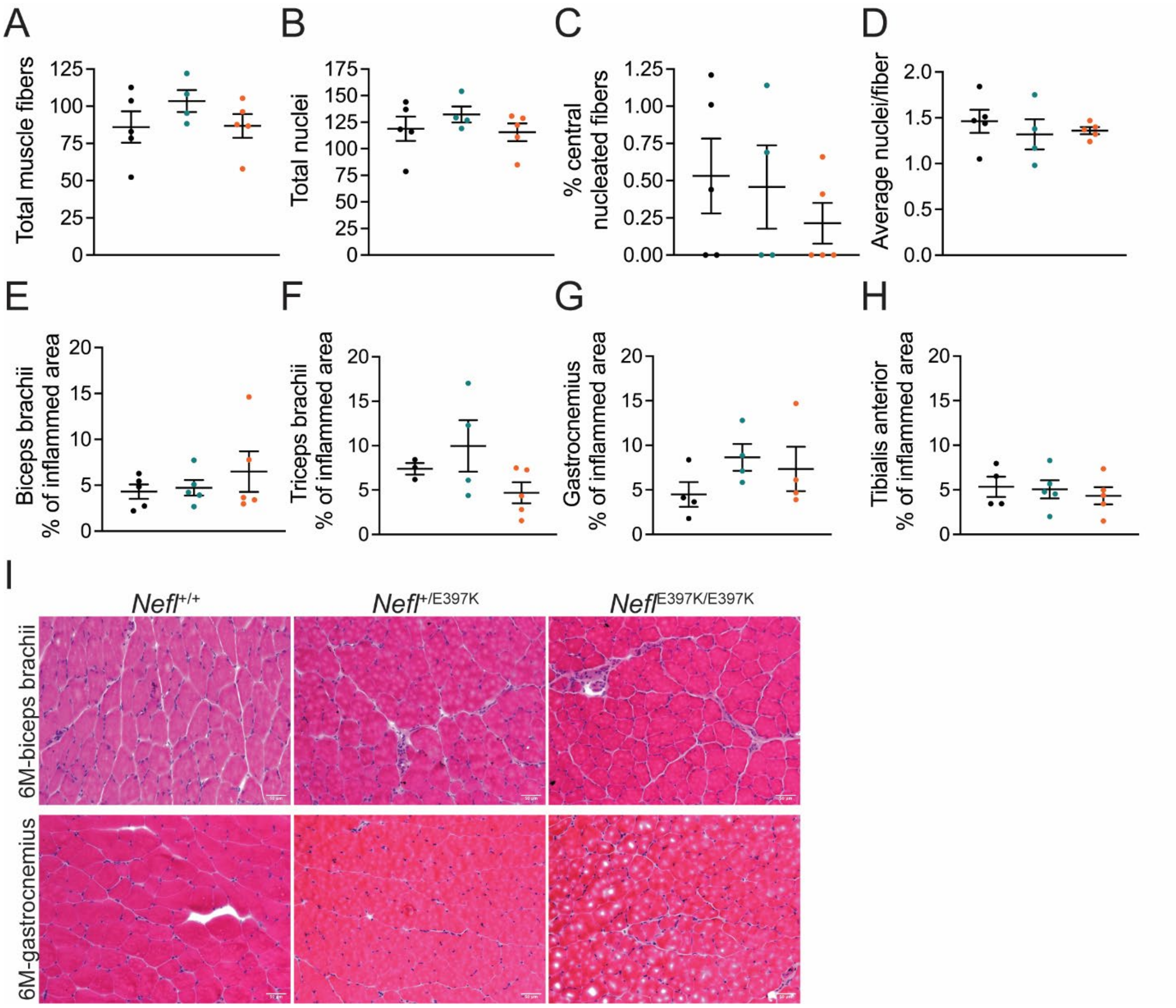

Supplementary Figure S5. Hematoxylin and eosin (H&E) staining of CMT2E mice. Cross sections of biceps brachii, triceps brachii, gastrocnemius, and tibialis anterior muscles of *Nefl*$^{+/+}$ (black), *Nefl*$^{+/E397K}$ (teal), and *Nefl*$^{E397K/E397K}$ (orange) were stained with H&E. (A) Total muscle fibers in the gastrocnemius muscle at six months (*Nefl*$^{+/+}$=86.0, *Nefl*$^{+/E397K}$=102.5, *Nefl*$^{E397K/E397K}$=86.8). (B) Total muscle fiber nuclei in the gastrocnemius muscle at six months (*Nefl*$^{+/+}$=118.8, *Nefl*$^{+/E397K}$=132.8, *Nefl*$^{E397K/E397K}$=115.5). (C) Percentage of central muscle fibers in the gastrocnemius muscle at six months (*Nefl*$^{+/+}$=0.53, *Nefl*$^{+/E397K}$=0.54, *Nefl*$^{E397K/E397K}$=0.21). (D) Average nuclei per muscle fiber in the gastrocnemius muscle at six months (*Nefl*$^{+/+}$=1.5, *Nefl*$^{+/E397K}$=1.3, *Nefl*$^{E397K/E397K}$=1.4). (E) Six months biceps brachii percentage of inflamed area of *Nefl*$^{+/+}$ (4.3%, N=5), *Nefl*$^{+/E397K}$ (4.7%, N=5), and *Nefl*$^{E397K/E397K}$ (6.5%, N=5). (F) Six months triceps brachii percentage of inflamed area of *Nefl*$^{+/+}$ (7.5%, N=3), *Nefl*$^{+/E397K}$ (10.7%, N=4), and *Nefl*$^{E397K/E397K}$ (4.7%, N=5). (G) Six months gastrocnemius percentage of inflamed area of *Nefl*$^{+/+}$ (4.5%, N=4), *Nefl*$^{+/E397K}$ (8.7%, N=4), and *Nefl*$^{E397K/E397K}$ (7.4%, N=4). (H) Six months tibialis anterior percentage of inflamed area of *Nefl*$^{+/+}$ (5.7%, N=4), *Nefl*$^{+/E397K}$ (5.1%, N=5), and *Nefl*$^{E397K/E397K}$ (4.3%, N=5). (I) Representative images of the H&E staining at six months (6M) of biceps brachii (top panel) and gastrocnemius (bottom panel) muscles. Two-way ANOVA with Dunnett multiple comparison test was used for statistical analyses. There was not significance between any of the analyzed parameters. Data points/dots represent mice. N=number of mice evaluated.
